# Validation of the Preoperative Score to Predict Postoperative Mortality (POSPOM) in Germany

**DOI:** 10.1371/journal.pone.0245841

**Published:** 2021-01-27

**Authors:** Yannik C. Layer, Jan Menzenbach, Yonah L. Layer, Andreas Mayr, Tobias Hilbert, Markus Velten, Andreas Hoeft, Maria Wittmann

**Affiliations:** 1 Department of Anaesthesiology and Intensive Care Medicine, University Hospital Bonn, Bonn, Germany; 2 Institute of Medical Biometrics, Informatics and Epidemiology (IMBIE), Medical Faculty, University of Bonn, Bonn, Germany; Technion - Israel Institute of Technology, ISRAEL

## Abstract

**Background:**

The Preoperative Score to Predict Postoperative Mortality (POSPOM) based on preoperatively available data was presented by Le Manach *et al*. in 2016. This prognostic model considers the kind of surgical procedure, patients' age and 15 defined comorbidities to predict the risk of postoperative in-hospital mortality. Objective of the present study was to validate POSPOM for the German healthcare coding system (G-POSPOM).

**Methods and findings:**

All cases involving anaesthesia performed at the University Hospital Bonn between 2006 and 2017 were analysed retrospectively. Procedures codified according to the French *Groupes Homogènes de Malades* (GHM) were translated and adapted to the German *Operationen- und Prozedurenschlüssel* (OPS). Comorbidities were identified by the documented International Statistical Classification of Diseases (ICD-10) coding. POSPOM was calculated for the analysed patient collective using these data according to the method described by Le Manach *et al*. Performance of thereby adapted POSPOM was tested using c-statistic, Brier score and a calibration plot. Validation was performed using data from 199,780 surgical cases. With a mean age of 56.33 years (SD 18.59) and a proportion of 49.24% females, the overall cohort had a mean POSPOM value of 18.18 (SD 8.11). There were 4,066 in-hospital deaths, corresponding to an in-hospital mortality rate of 2.04% (95% CI 1.97 to 2.09%) in our sample. POSPOM showed a good performance with a c-statistic of 0.771 and a Brier score of 0.021.

**Conclusions:**

After adapting POSPOM to the German coding system, we were able to validate the score using patient data of a German university hospital. According to previous demonstration for French patient cohorts, we observed a good correlation of POSPOM with in-hospital mortality. Therefore, further adjustments of POSPOM considering also multicentre and transnational validation should be pursued based on this proof of concept.

## 1. Introduction

In 2012 there was an estimate of 312 million surgical procedures performed worldwide, with increasing cases over the last years [1]. Although overall perioperative mortality had declined, complication rates are still high especially among elderly patients and those exhibiting various and severe comorbidities, leading to 4.2 million patients dying within 30 days of surgery worldwide per year. Numerous studies investigated perioperative death, revealing mortality rates ranging from 0.5% in the International Surgical Outcomes Study (ISOS, total in-hospital mortality) over 1.3% in the Vascular Events In Noncardiac Surgery Patients Cohort Evaluation (VISION, total postoperative 30-day mortality) up to 1.85% in the American College of Surgeons National Surgical Quality Improvement Program database (ACS-NSQIP, total postoperative 30-day mortality) [2–6]. Therefore, it is imperative to further control perioperative mortality, utilizing evidence-based best practice. In this regard, clinical decision making as well as risk communication in surgery and anaesthesia both rely on the precise prognosis of perioperative mortality [7].

Various tools have been developed and validated to help physicians weighing risks and benefits especially of elective surgery. However, many of those scoring systems are either time-consuming and therefore difficult to implement into clinical routine, or they do have considerable limitations. While some scores solely focus on the surgical procedure itself, others only evaluate the patients’ physical status and comorbidities [8]. One prominent example is the widely acknowledged and commonly used American Society of Anesthesiologists Physical Status score (ASA-PS) [9]. Based on the individual assessment of the anaesthesiologist, patients are allocated to six major risk groups on a rather subjective judgement [10]. A further limitation results from ASA Score neither considering the kind of surgery nor patient's age. Other scoring systems such as the Physiological and Operative Severity Score for the enumeration of Mortality (POSSUM) and its modifications rely on intraoperative details that cannot be obtained prior to surgery [11, 12].

In 2016, Le Manach *et al*. presented the Preoperative Score to Predict Postoperative Mortality (POSPOM) [13]. The authors’ aim was to develop an objective, yet easy-to-use score exclusively relying on preoperatively available information. The score is derived from the types of surgical procedures requiring anaesthesia, covering emergency as well as elective operations. In addition, patients' age and significant comorbidities contribute to the POSPOM value. The latter is an individual score value, indicating the patient’s risk for postsurgical in-hospital death. The POSPOM was derived from data of hospitals all over France performing at least 500 surgical procedures in adults within the year 2010. By involving a total of more than 5.5 million patient data sets in either a derivation or validation cohort, Le Manach *et al*. generated and validated a convincing prognostic model.

Despite the general availability of the required data, the POSPOM has not been validated for the German healthcare system and therefore cannot be routinely used in Germany. It was our aim to enable the application of the POSPOM by adapting it to the national coding system further referred to as G-POSPOM and to validate its prognostic power on data of a large patient sample from a German university hospital.

## 2. Materials and methods

Adaptation of the POSPOM to the German healthcare coding system was performed by retrospective calculation, based on patient data extracted from the anonymized data set following *§21 Krankenhausentgeltgesetz* (KHEntgG, German hospital fees act), which is used for billing purposes [14].

Ethical approval for this study was waived by the Ethics Committee of the University Hospital Bonn (084/20) because according to the Professional Code of Conduct of the Medical Association of North Rhine-Westphalia §15 the approval is not necessary for a retrospective analysis [15]. This waiver also waived the requirement for informed consent.

### Patient selection

All surgical procedures or interventions involving anaesthesia performed on adult patients (at least 18 years of age) at the University Hospital Bonn, Germany, between January 1st 2006 and December 31st 2017 were identified. Data required for the calculation of the POSPOM were collected by reviewing the institutional §21 KHEntgG electronic database, which was accessed by the authors on July 4th 2018. Age, hospital-intern-tag (anonymized), OPS-Codes, ICD-Codes, dates of stay and cause of discharge were extracted. Random samples were checked with the hospital information system. Investigated endpoints were patients' discharge or in-hospital death. Death after hospital discharge was not taken into account. The POSPOM variables are included in S1 Table.

The French *Groupes Homogènes de Malades* (GHM) coding system as the national equivalent to the German *Diagnosis Related Groups* (DRG) contains both information on diseases and comorbidities according to the *Classification Internationale des Maladies* (CIM-10, corresponding to the *International Statistical Classification of Diseases* 10th Revision [ICD-10]) and on surgical and interventional procedures according to the *Classification Commune des Actes Médicaux* (CCAM). GHM codes used by Le Manach *et al*. for the respective CCAM had to be translated into the German Surgery and Procedure coding system (*Operationen- und Prozedurenschlüssel* (OPS)) [16, 17]. French index surgeries were manually assigned to German OPS equivalents. Inconclusive surgical procedures were reviewed by specialist surgeons of the corresponding departments. Patients that underwent multiple surgeries had their first index surgery assigned. In case of a patient having more than one relevant index procedure encoded at the same time, we assigned the surgery scoring the most POSPOM points. Comorbidities were coded using ICD-10. Due to its similarity to the French system, ICD codes were reviewed but not modified [18]. Afterwards, the POSPOM values were calculated as shown by Le Manach *et al*. (S1 Table). Patients were excluded from analyses if any of the POSPOM variables were not attainable for a patient during the investigation period.

This report complies with the Strengthening the Reporting of Observational Studies in Epidemiology (STROBE) guidelines for observational cohort studies as stated in S2 Table [19].

### Statistical analysis

Statistical performance and accuracy of the prognostic model was measured testing discrimination and calibration. Discrimination was checked and visualized by a receiver operating characteristic (ROC) analysis and calculation of its area under the curve (AUC), also termed c-statistic [20]. Possible values of the AUC vary from 0.5 (no predictive ability) to 1.0 (perfect predictive ability). The Brier score assesses the overall accuracy (discrimination and calibration), ranging from 0 (implying perfect prediction) to 1 (worst possible prediction). Calibration was visualized via a calibration plot in which a gradient of 1 with the diagonal crossing the origin equals perfect calibration.

All analyses were carried out using the software R (Version 3.5.0 (http://www.r-project.org), last date accessed: January 23, 2020) under creative common license and the affiliated packages ggplot2, dplyr, and pROC [21–23].

## 3. Results

**Fig 1:**
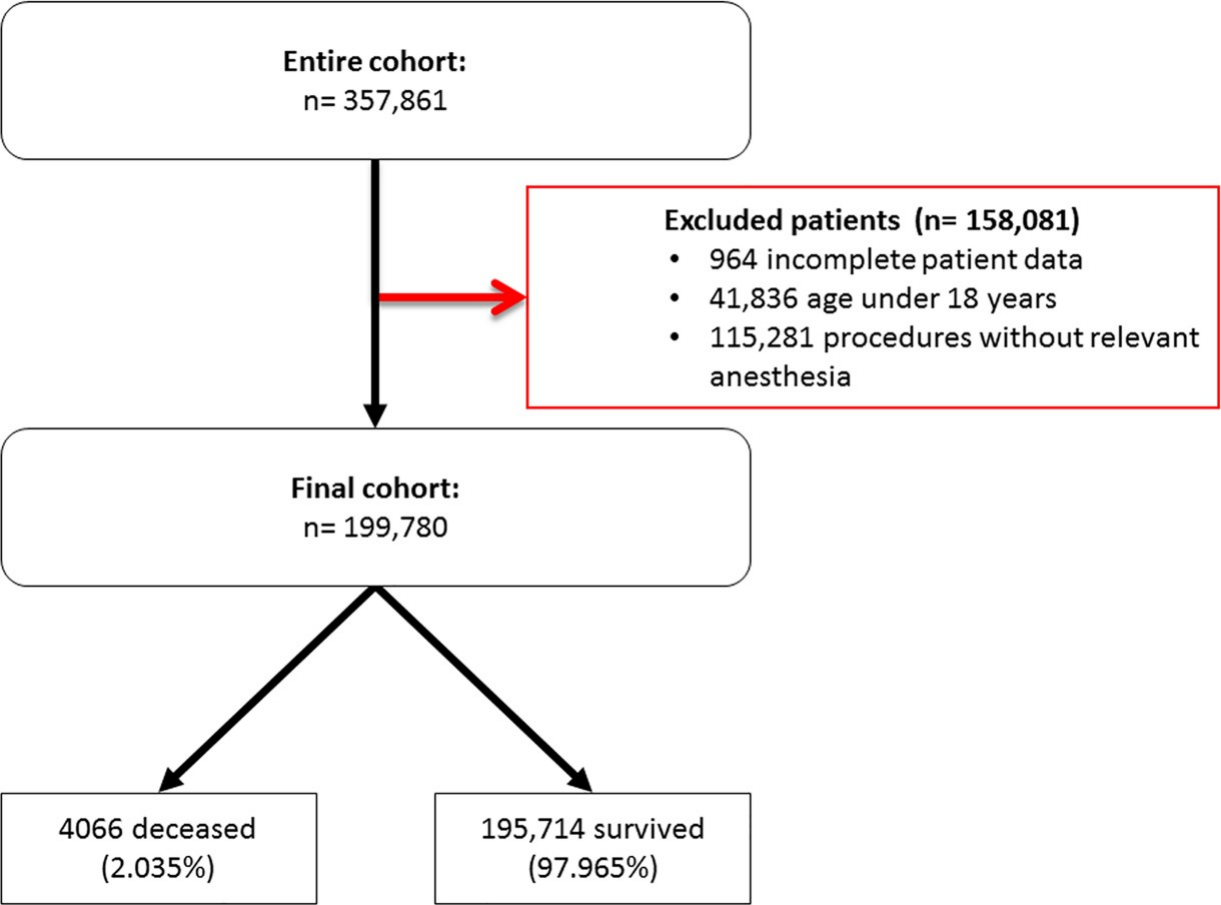
Patient flow chart. Procedures without relevant anaesthesia include mainly patients of intensive care, patients who received electroconvulsion therapy or minor interventions as biopsies.

A total of 357,861 surgical cases during the time period between January 1st 2006 and December 31st 2017 were identified from the institutional data base at the University Hospital Bonn. Of those cases, 115,281 had no index procedures relevant for the POSPOM and were therefore excluded from further analyses. These were mainly interventions during intensive care therapy, patients undergoing electroconvulsive treatment, or patients having received minor interventions such as biopsies. 41,836 cases were excluded as the patients were younger than 18 years. Finally, 964 cases showed an incomplete dataset with at least one missing relevant information, resulting in a POSPOM not being able to be calculated. Fig 1 shows the patient flow chart diagram.

We included a total of 199,780 patient cases along with 4,053 in-hospital deaths into our study, resulting in a hospital mortality of 2.04% (95% CI 1.97 to 2.09%). In our cohort 98,376 patients (49.24%) were female and 101,394 patients (50.75%) were male with an in-hospital mortality of 1.73% and 2.33% respectively. The highest score value observed was 49 POSPOM-Points. Mean POSPOM value in our cohort was 18.18 (SD 8.11), the median POSPOM was 18 points. Women had a mean POSPOM of 16.96 (SD 7.90) and men a higher mean of 19.35 (SD 8.14). Mean age was 56.33 (SD 18.59) years, and the median age was 59 years. Mean age of male patients was 58.38 (SD 17.45) years, while the median age was 61 years. Woman had a mean age of 54.25 (SD 19.47) years and a median age of 55 years. In total, 3,631,032 POSPOM points were applied to our patient cohort. Of those, 354,229 points (9.76%) were scored for comorbidities, 1,531,337 points (42.17%) for age, and 1,745,466 points (48.07%) were scored for surgeries.

**Fig 2:**
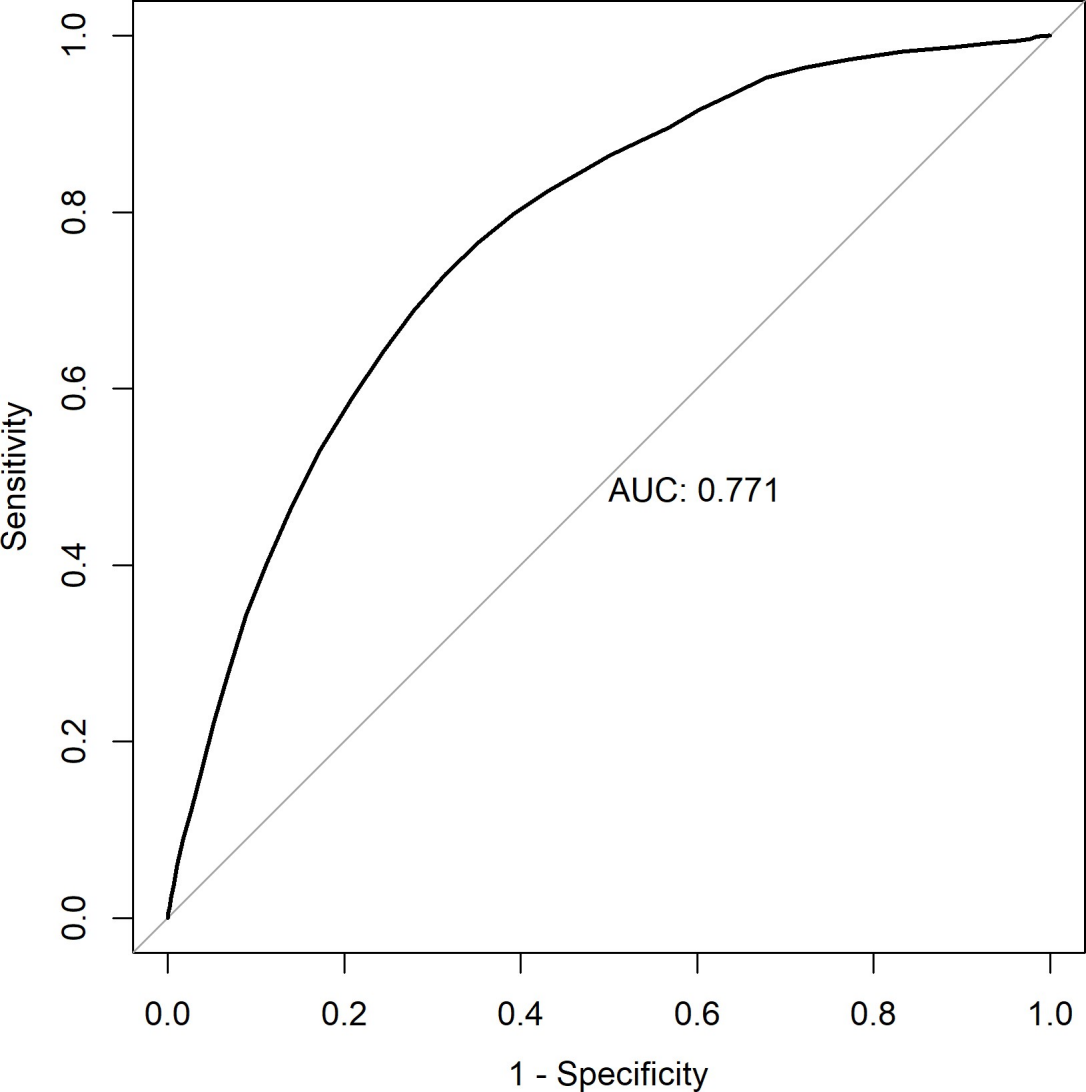
Receiver operating characteristic (ROC) curve. The ROC curve shows the false positive rate on the X-axis and the true positive rate on the Y-axis. The optimum is an area under the curve (AUC) of 1.

**Fig 3:**
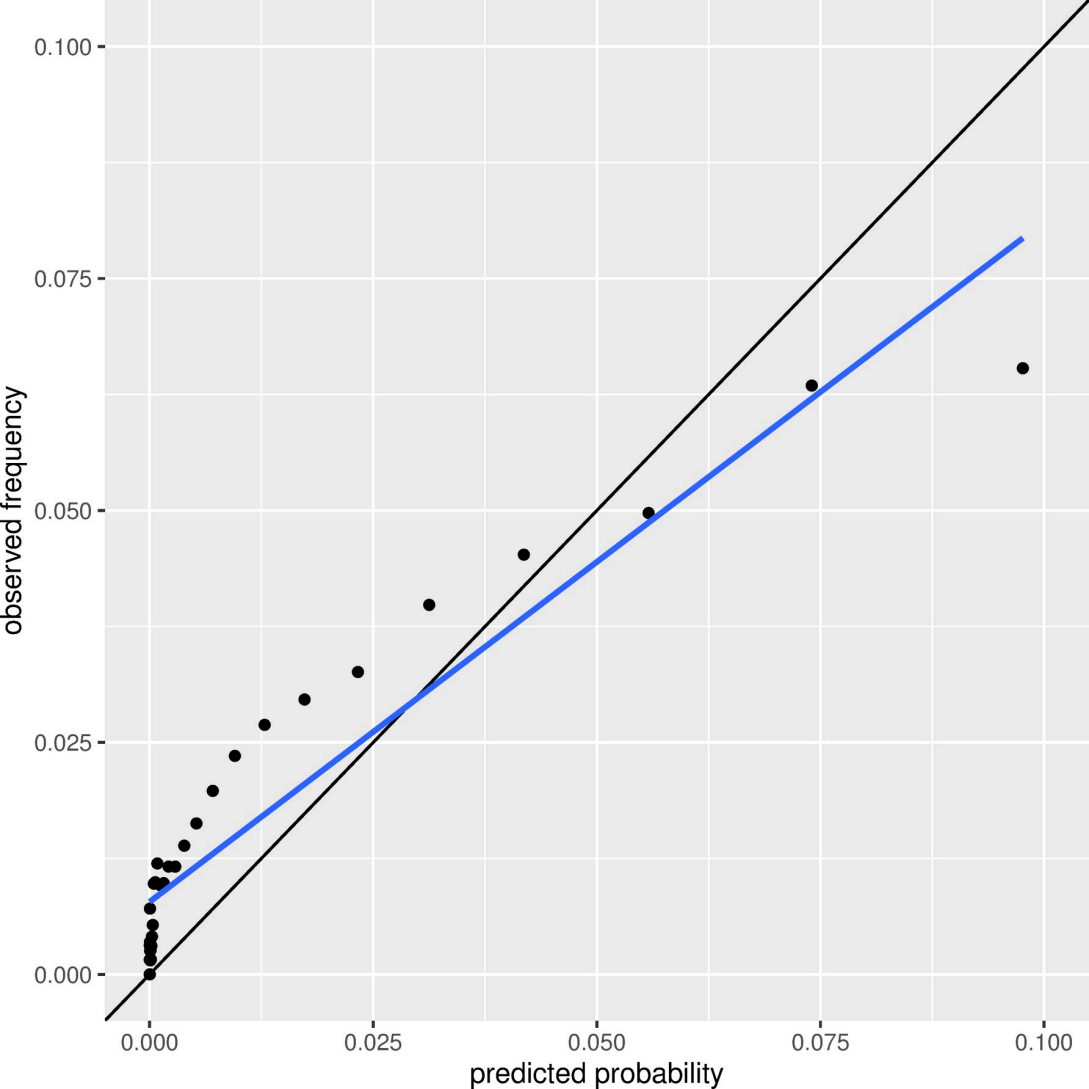
Calibration plot with predicted and observed in-hospital mortality for POSPOM. The bisectrix equals perfect calibration. Black dots represent the observed values. The blue line is the line of best fit using linear regression. Grey shade shows the confidence interval.

In our study population, the accordingly adapted G-POSPOM system showed a c-statistic of 0.771 and a Brier score of 0.021. Fig 2 displays the area under the curve (AUC) for the receiver operating characteristics analysis (ROC). Fig 3 shows the calibration plot. The plot indicates an underestimation of mortality in the area below 5% mortality and an overestimation of mortality above this threshold.

**Fig 4:**
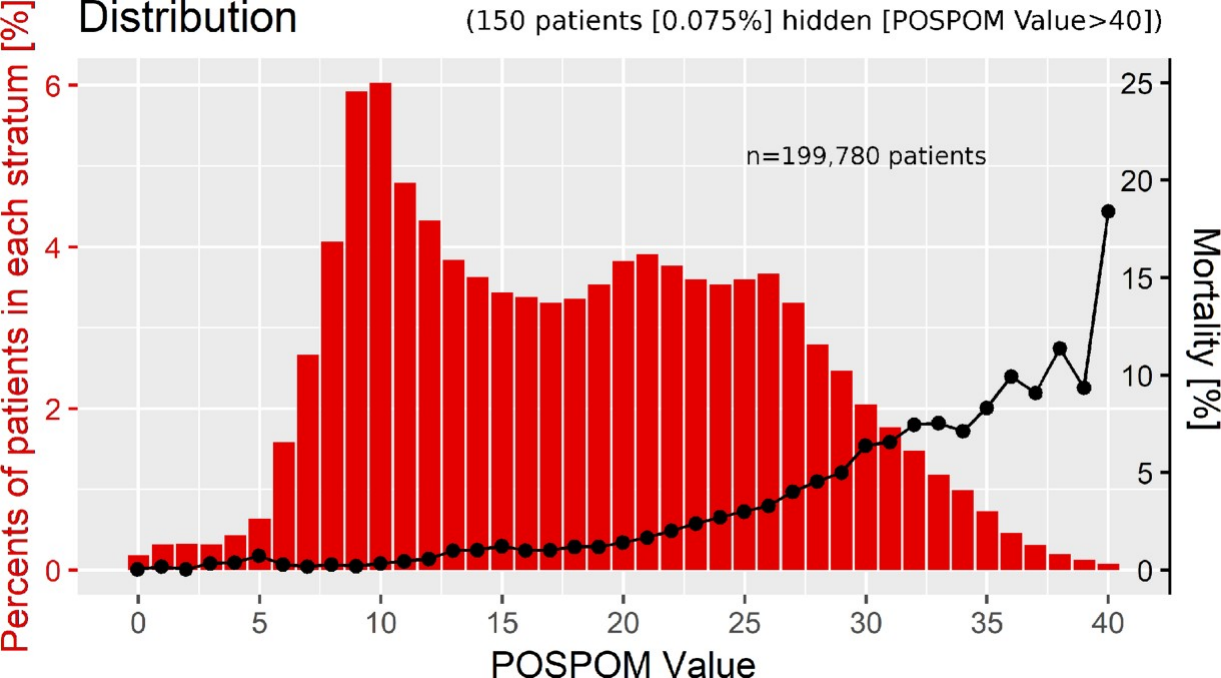
Distribution of the POSPOM scores in the cohort of Bonn University Hospital (n = 199,780) in relation to the observed in-hospital mortality rate at each POSPOM value between 0 and 40.

**Fig 5:**
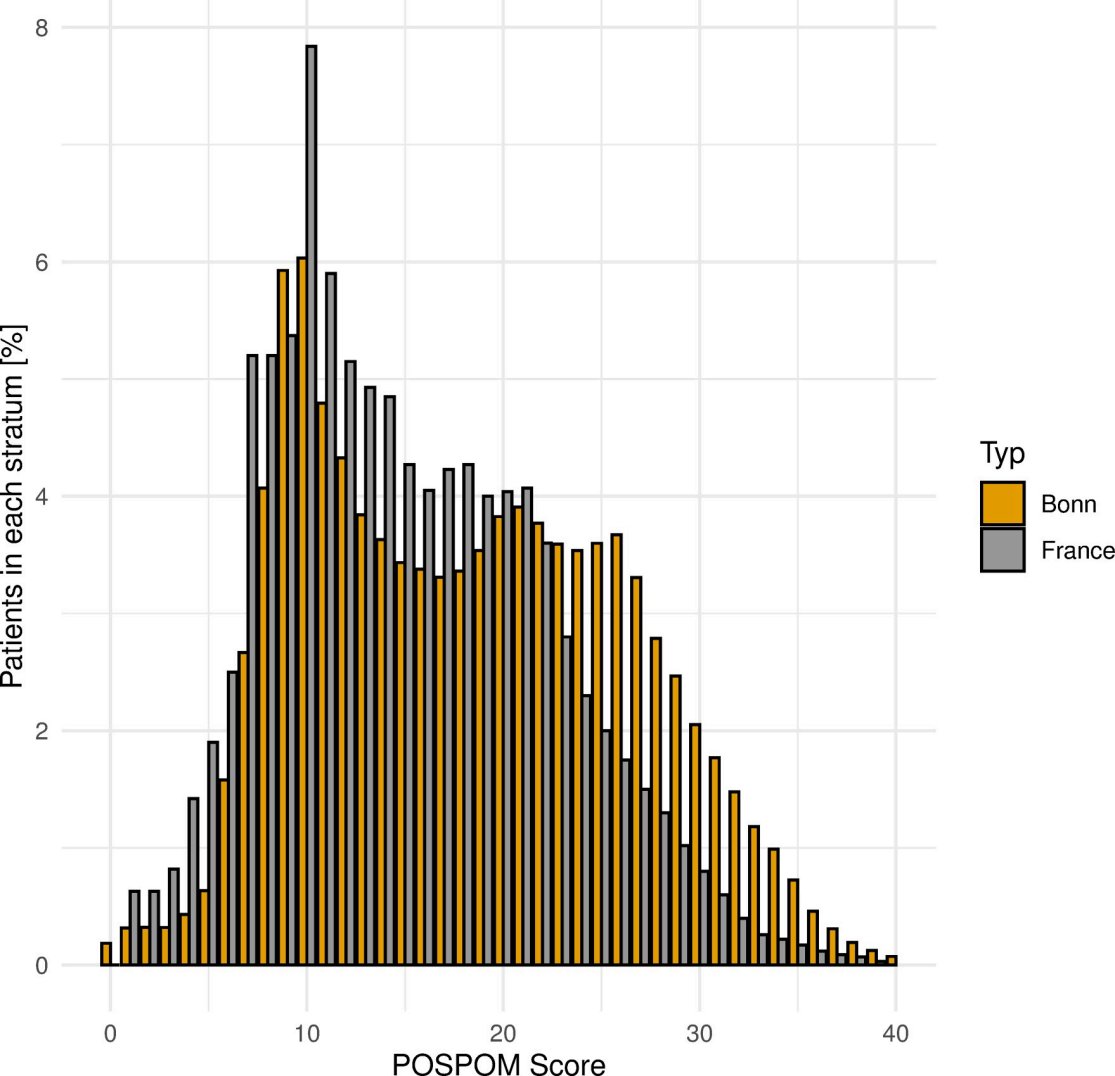
Distribution of the POSPOM scores in the cohort of Bonn University Hospital (orange bars, n = 199,780) compared to POSPOM validation cohort (grey bars, n = 2,789,932).

**Fig 6:**
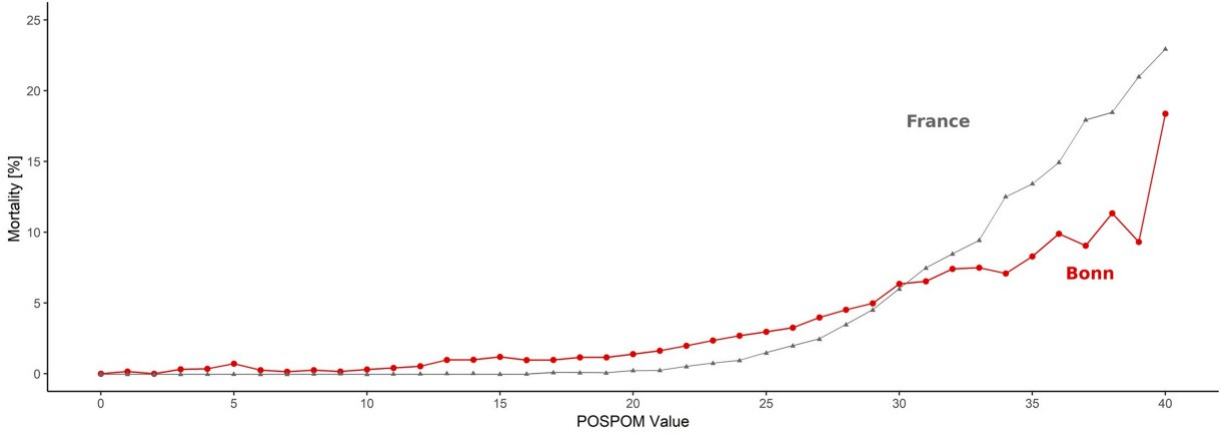
Observed in-hospital mortality rate at each POSPOM value between 0 and 40 in the cohort of Bonn University Hospital (red line, n = 199,780) compared to POSPOM validation cohort (grey line, n = 2,789,932).

Fig 4 shows the distribution of the POSPOM values at the University Hospital Bonn in relation to the observed in-hospital mortality. Fig 5 compares this distribution to the one in the French population as described by Le Manach *et al*. We also compared these two groups regarding in-hospital mortality (Fig 6). In our cohort, 22.49% of the patients scored 10 points or less, while 31.5% of the French study sample received such a low score. 25.26% of the patients in the university hospital of Bonn scored 25 points or more, compared to approximately 10% of the patients in the French cohort. Mortality in our study population was 0.31% at a score of 10 POSPOM points, 1.39% at 20 POSPOM points, 6.35% at 30 POSPOM points and 18,37% at 40 POSPOM points, compared to 0%, 0.2%, 6% and 23%, respectively, in the French study population of Le Manach *et al*. (S3 Table)

## 4. Discussion

Our study demonstrates that the POSPOM, originally derived from French data, can successfully be transferred into other national healthcare systems, in our case by matching to the German OPS coding. Thereby the adapted G-POSPOM may accurately predict postoperative in-hospital mortality.

With an increased incidence of perioperative complications in defined groups of patients and procedures, accurate risk prediction becomes crucial not only for communicating the imminent risk of surgery to patients, but also for clinical decision making and risk management. There will always be deviation in a subjective score such as the ASA and added objective scores can help to detect patients at risk and prevent misjudgement especially in cases of uncertainty. G-POSPOM does not substitute clinical decision making, but it might be a helpful tool to add an additional view to the clinical decision.

While various established risk assessment scores appraise either solely the patient or the procedure, Le Manach *et al*. developed an easy-to-implement scoring system covering both surgery as well as patient-specific variables from the French National Hospital Discharge Data Base (NHDBB) system to provide a precise prediction of the patient’s individual risk for postoperative death. To use the potential of the POSPOM as a valuable tool in clinical decision making also outside France, it needs to be adapted to the corresponding national coding system.

Comparing to the excellent discrimination (c-statistic: 0.944) and accuracy (Brier score: 0.004) achieved by Le Manach *et al*. in their derivation cohort and in their validation cohort (c-statistic: 0.929 and Brier score: 0.005) we did not get equally strong but still convincing findings (c-statistic of 0.771 and a Brier score of 0.021) from our patient sample [13].

Several issues make the POSPOM adaptation as well as external validation challenging. These are, among others, the transfer of the different national codifications of procedures, data quality, the study design (unicentric vs. multicentric, elective vs. emergency interventions) and differences in specialization and expertise of surgeons as well as perioperative care specialists.

Codification as well as classification of procedures differ between the individual healthcare systems and therefore need to be adapted when transferring the POSPOM. Accordingly, the POSPOM had to be modified for the German codification system of operations and procedures, as this differs from the French classification for medical procedures, the CCAM, that has been used for the development of the POSPOM by Le Manach *et al*. Modification was first based on the authors´ assessment and secondly reviewed by specialists of the corresponding surgical compartments. A reduction in predictive and discriminative power of the G-POSPOM is therefore possible, making further validation of our adaptation mandatory.

With the perspective of the development of the International Classification of Health Interventions (ICHI) it may be less difficult to compare data and thus use prognostic scores and tools like the POSPOM internationally [24]. Especially combined with well-proven and common scores such as ASA this could offer an opportunity to further improve patient safety.

It seems challenging to get complete patient datasets without any missing codification information, a limitation that also seems to apply to the POSPOM cohort of Le Manach *et al*. Regarding the ICD diagnoses used to record comorbidities in the original French cohorts, diabetes mellitus or arterial hypertension seem to be quite underrepresented compared to average populations. This was also seen in our validation group, just as in the French derivation and validation cohorts [14]. Furthermore, codes or pre-existing diagnoses that do not influence medical management or treatment of patients should not be coded in the German DRG system [25]. Even though data quality seems to have improved over the years, access to ‘perfect’ data quality is highly unlikely, as it likewise differs not only between hospitals but also wards and even individual medical staff [26]. However, this may not necessarily affect the general applicability and validity of the POSPOM, as the score was derived facing the same problem. Still, used in different hospitals with unsteady data quality deviating appreciable from the overall French quality, it might inhibit the implementation of the POSPOM. Only 9.76% of the applied POSPOM points referred to comorbidities and therefore only had a small impact, a bad data quality of comorbidities could thus have a minor effect on the score but rather effect its subtleties. However, precision of the scoring system might be affected.

The present study is a retrospective, single-centered analysis integrating data recorded over a period of 11 years and is therefore limited in its comparability to the French multi-centre derivation cohort assessed within one year. In 2016, the University Hospital Bonn had an average case mix index of 1.76 compared to a German average of 1.1, indicating that patients with more severe and multiple comorbidities are treated to a higher degree. Therefore, our study population differs from smaller, highly specialized hospitals or hospitals providing primary healthcare [27, 28]. Severely ill or injured patients might therefore be overrepresented and others underrepresented, thus worsening overall outcome and explaining a more moderate predictive power of the adapted G-POSPOM.

In consequence, we observed a significantly higher postoperative mortality rate of 2%, compared to 0.5% in the French derivation and validation cohorts. Furthermore, our reported mortality rate is higher than described by the ISOS study, most likely due to a higher percentage of critically ill or severely injured patients [2]. A higher percentage of the patients of our cohort scored more than 25 POSPOM points, compared to the average French patient cohorts. On the other hand, a lower percentage scored less, implying older patients exhibiting more comorbidities and receiving complex surgeries. Even though more patients died scoring 10–20 POSPOM points, the University Hospital Bonn seemed to have a better performance regarding higher POSPOM values. This might be explained by increased expertise regarding critically ill patients and thus possibly a lower rate of death due to postoperative adverse events, known as failure to rescue [29]. The decreasing mortality at the score values of 34, 37 and 39 POSPOM points in Bonn may be explained by an accumulation of cardiac surgery patients which had an exceedingly low mortality rate.

The POSPOM does not distinguish between emergency or elective surgery. However, seen from the clinician’s point of view, there is obviously a considerable difference between elective and emergency patients, regarding mortality rate. Therefore, it might be reasonable to take the treatment mode such as elective, urgent or even emergent into account for the prognostic model and thus for scoring.

Up to now, just a few studies validated the POSPOM in different national medical systems. One validation study was performed in Germany, based on a patient cohort of 1083 cases limited to the procedure of radical cystectomy [30]. The authors showed a good discriminative accuracy, but experienced a mortality lower than the predicted one. However, there are some differences of that study compared to our analysis. The data base of the respective study did not provide complete information on all comorbidities required to fully adapt the POSPOM. Furthermore, the authors used a rather small study population and focused just on one procedure. Last, the end points were 30-day mortality and 90-day mortality instead of in-hospital mortality. Therefore, it provides only limited information on the performance of the POSPOM in the German healthcare system. Another validation was performed using data of 782 patients, stating only a poor performance for a cohort of geriatric patients scheduled for hip fracture surgery [31]. In Denmark, a study comprising 979 patients with major emergency abdominal surgery reported a good discrimination but poor calibration for the POSPOM [32]. Last, a study conducted in Portugal on 833 patients admitted to intensive care after open vascular surgery reported a better prediction rate for the POSPOM compared to APACHE or SAPS [33]. The attempts to validate the POSPOM in different other countries emphasises the need for a simple score to predict postoperative mortality worldwide. However, all these studies focused on just one single procedure combined with age and comorbidities and had some other limitations that need to be addressed. In contrast to our analysis, numbers of cases were rather low.

Different to the POSPOM, the NSQIP Surgical Risk Calculator refers to the 30-day outcome in terms of mortality, morbidity and complications [5]. The resulting prognostic model showed excellent performance for mortality (c-statistic: 0.944; Brier score: 0.011), morbidity (c-statistic: 0.816, Brier score: 0.069), and 6 additional complications (c-statistic: > 0.8). Advantage of the web-based application is the possibility to be used by both physicians and patients before planning surgical treatments. However, the survey and assessment are more likely to be fully applicable by doctors, which may cause uncertainty for some patients. The required variables appear to be regularly obtainable preoperatively in clinical routine. Whether they are included in the individual data sets for retrospective validation studies or whether prospective studies for validation are required remains to be clarified. Limiting to hospitals that participated in the charged NSQIP and the investigation in the American healthcare system, which differs significantly from many European systems, hinders the applicability to European populations and the comparability with the POSPOM.

In summary, in our analysis the G-POSPOM proved to be a valuable score to predict in-hospital mortality in patients undergoing elective as well as emergency surgery. Using preoperatively available data from a retrospective data base record, we could demonstrate that the POSPOM is applicable to the German healthcare system as well as to the patient population of a single-centre university hospital. However, further multicentric validation is mandatory as the patient population of a single university hospital most likely does not represent the overall German healthcare system. Recalibration and prospective study designs could help to further improve the POSPOM’s applicability to the German patient population as much as to other national healthcare systems.

## Supporting information

S1 Table (DOCX)

S2 Table STROBE statement—checklist of items that should be included in reports of cohort studies. (DOC)

S3 Table (DOCX)

S4 Table (DOCX)

S5 Table (DOCX)

S6 Table (DOCX)

## References

[1] Weiser TG, Haynes AB, Molina G, Lipsitz SR, Esquivel MM, Uribe-Leitz T, et al Estimate of the global volume of surgery in 2012: an assessment supporting improved health outcomes. Lancet 2015; 385(Suppl. 2), S11 10.1016/S0140-6736(15)60806-6 26313057

[2] International Surgical Outcomes Study Group. Global patient outcomes after elective surgery: prospective cohort study in 27 low-, middle-and high-income countries. Br J Anaesth 2016; 117:601–609. 10.1093/bja/aew316 27799174 PMC5091334

[3] Devereaux PJ, Chan MT, Alonso-Coello P; Vascular Events In Noncardiac Surgery Patients Cohort Evaluation (VISION) Study Investigators: Association Between Postoperative Troponin Levels and 30-Day Mortality Among Patients Undergoing Noncardiac Surgery. JAMA 2012; 307(21):2295–2304. 10.1001/jama.2012.5502 22706835

[4] Glance LG, Lustik SJ, Hannan EL, Osler TM, Mukamel DB, Qian F, et al The surgical mortality probability model. Ann Surg 2012; 255(4):696–702 10.1097/SLA.0b013e31824b45af 22418007

[5] Bilimoria KY, Liu Y, Paruch JL, Zhou L, Kmiecik TE, Ko CY, et al Development and Evaluation of the Universal ACS NSQIP Surgical Risk Calculator: A Decision Aide and Informed Consent Tool for Patients and Surgeons. J Am Coll Surg 2013; 217(5):833–842.e3. 10.1016/j.jamcollsurg.2013.07.385 24055383 PMC3805776

[6] Bainbridge D, Martin J, Arango M, Cheng D; Evidence-based Peri-operative Clinical Outcomes Research (EPiCOR) Group: Perioperative and anaesthetic-related mortality in developed and developing countries: a systematic review and meta-analysis. Lancet 2012; 380 (9847) pp. 1075–1081 10.1016/S0140-6736(12)60990-8 22998717

[7] Wijeysundera DN. Predicting outcomes: Is there utility in risk scores? Can J Anesth/J Can Anesth 2016; 63: 148 10.1007/s12630-015-0537-2 26670801

[8] Lee TH, Marcantonio ER, Mangione CM, Thomas EJ, Polanczyk CA, Cook EF, et al Derivation and Prospective Validation of a Simple Index for Prediction of Cardiac Risk of Major Noncardiac Surgery. Circulation 1999; 100(10):1043–9. 10.1161/01.cir.100.10.1043 10477528

[9] Hackett NJ, De Oliveira GS, Jain UK, Kim JY. ASA class is a reliable independent predictor of medical complications and mortality following surgery. Int J Surg 2015; 18:184–90. 10.1016/j.ijsu.2015.04.079 25937154

[10] Sankar A, Johnson SR, Beattie WS, Tait G, Wijeysundera DN: Reliability of the American Society of Anesthesiologists physical status scale in clinical practice. Br J Anaesth 2014; 113(3):424–432. 10.1093/bja/aeu100 24727705 PMC4136425

[11] Copeland GP, Jones D, Walters M. POSSUM: a scoring system for surgical audit. Br J Surg 1991; 78(3):355–60. 10.1002/bjs.1800780327 2021856

[12] Prytherch DR, Whiteley MS, Higgins B, Weaver PC, Prout WG, Powell SJ. POSSUM and Portsmouth POSSUM for predicting mortality Physiological and Operative Severity Score for the enUmeration of Mortality and morbidity. Br J Surg 1998; 85(9):1217–20. 10.1046/j.1365-2168.1998.00840.x 9752863

[13] Le Manach Y, Collins G, Rodseth R, Le Bihan-Benjamin C, Biccard B, Riou B et al Preoperative Score to Predict Postoperative Mortality (POSPOM): Derivation and Validation. Anesthesiology 2016; 124(3):570–579. 10.1097/ALN.0000000000000972 26655494

[14] §21 Krankenhausentgeltgesetz Available at: https://www.gesetze-im-internet.de/khentgg/__21.html Accessed January 23, 2020

[15] Berufsordnung für die nordrheinischen Ärztinnen und Ärzte. Available at: https://www.aekno.de/aerzte/berufsordnung Accessed January 23,2020

[16] Manuel des Groupes Homogènes de Maladies; Ministère de la Santé et des Sports, France. Available at: https://www.atih.sante.fr/sites/default/files/public/content/2708/volume_1.pdf Accessed January 23, 2020

[17] Operationen- und Prozedurenschlüssel Version 2018. Available at: https://www.dimdi.de/static/de/klassifikationen/ops/kode-suche/opshtml2019/ Accessed January 23,2020

[18] Internationale statistische Klassifikation der Krankheiten und verwandter Gesundheitsprobleme 10. Revision German Modification Version 2019. Available at: https://www.dimdi.de/static/de/klassifikationen/icd/icd-10-gm/kode-suche/htmlgm2019/ Accessed January 23,2020

[19] von Elm E, Altman DG, Egger M, Pocock SJ, Gotzsche PC, Vandenbroucke JP; STROBE Initiative. The strengthening the reporting of observational studies in epidemiology (STROBE) statement: guidelines for reporting observational studies. Int J Surg 2014; 12(12):1495–1499. 10.1016/j.ijsu.2014.07.013 25046131

[20] Cook NR. Statistical evaluation of prognostic versus diagnostic models: beyond the ROC curve. Clin Chem 2008; 54:17–23. 10.1373/clinchem.2007.096529 18024533

[21] Wickham H. ggplot2: Elegant Graphics for Data Analysis. Springer-Verlag New York, 2016.

[22] Wickham H, François R, Henry L, Müller K. dplyr: A Grammar of Data Manipulation. R package version 0.7.6. 2018 Available at: https://CRAN.R-project.org/package=dplyr Accessed January 31,2020

[23] Robin X, Turck N, Hainard A, Tiberti N, Lisacek F, Sanchez JC, et al pROC: an open-source package for R and S+ to analyze and compare ROC curves. BMC Bioinformatics 2011, 12, p. 77 10.1186/1471-2105-12-77 21414208 PMC3068975

[24] Zaiss A, Dauben HP. International classification of health interventions. A balancing act between the demands of statistics and reimbursement. Bundesgesundheitsblatt 2018; 61:788. 10.1007/s00103-018-2747-6 29808283

[25] DEUTSCHE KODIERRICHTLINIEN: Allgemeine und Spezielle Kodierrichtlinien für die Verschlüsselung von Krankheiten und Prozeduren Version 2019 Available at: https://www.dkgev.de/fileadmin/default/Mediapool/2_Themen/2.4._Medizin_und_Wissenschaft/2.4.2._Medizinische_Klassifikationen/2.4.2.3._Kodierrichtlinien/DKR_2019.pdf Accessed January 28,2020

[26] Heywood NA, Gill MD, Charlwood N, Brindle R, Kirwan CC. Improving accuracy of clinical coding in surgery: collaboration is key. Journal of Surgical Research 2016, Volume 204, Issue 2, 490–495 10.1016/j.jss.2016.05.023 27565087

[27] Statistisches Bundesamt: Fachserie 12 Reihe 6.4 Gesundheit. Available at: https://www.destatis.de/DE/Themen/Gesellschaft-Umwelt/Gesundheit/Krankenhaeuser/Publikationen/Downloads-Krankenhaeuser/fallpauschalen-krankenhaus-2120640167004.pdf?__blob=publicationFile Accessed February 6,2020

[28] Universitätsklinikum Bonn Geschäftsbericht 2017. Available at: https://www.ukbonn.de/C12582D3002FD21D/vwLookupDownloads/Inhalt_Geschaeftsbericht_2017_2018-09-04-Ansicht.pdf/$FILE/Inhalt_Geschaeftsbericht_2017_2018-09-04-Ansicht.pdf Accessed February 6,2020

[29] Portuondo JI, Shah SR, Singh H, Massarweh NN. Failure to Rescue as a Surgical Quality Indicator: Current Concepts and Future Directions for Improving Surgical Outcomes. Anesthesiology 2019; 131(2):426–437 10.1097/ALN.0000000000002602 30860985

[30] Froehner M, Koch R, Hubler M, Heberling U, Novotny V, Zastrow S, et al Validation of the Preoperative Score to Predict Postoperative Mortality in Patients Undergoing Radical Cystectomy. European Urology Focus 2019; 5 (2), pp. 197–200 10.1016/j.euf.2017.05.003 28753894

[31] Niessen R, Bihin B, Gourdin M, Yombi JC, Cornu O, Forget P. Prediction of postoperative mortality in elderly patient with hip fractures: a single-centre, retrospective cohort study. BMC Anesthesiol 2018; 18, 183 10.1186/s12871-018-0646-x 30509182 PMC6278082

[32] Juul S, Kokotovic D, Degett TH, Oreskov JO, Ekeloef S, Gögenur I, et al Validation of the preoperative score to predict postoperative mortality (POSPOM) in patients undergoing major emergency abdominal surgery. Eur J Trauma Emerg Surg 2019 10.1007/s00068-019-01153-x 31161251

[33] Reis P, Lopes AI, Leite D, Moreira J, Mendes L, Ferraz S, et al Predicting mortality in patients admitted to the intensive care unit after open vascular surgery. Surg Today 2019; 49, 836–842 10.1007/s00595-019-01805-w 30968224

