# Supplementary material for: Validation of the Preoperative Score to Predict Postoperative Mortality (POSPOM) in Germany
Source: PLoS One. 2021 Jan 27;16(1):e0245841. doi: 10.1371/journal.pone.0245841 (PMC7840059; doi:10.1371/journal.pone.0245841)
Supplement: S2 Table — (DOC) [file pone.0245841.s002.doc]

STROBE Statement—Checklist of items that should be included in reports of ***cohort studies***

|  | Item No | Recommendation | Text Excerpt |
| --- | --- | --- | --- |
| **Title and abstract** | 1 | (*a*) Indicate the study’s design with a commonly used term in the title or the abstract | “All cases involving anaesthesia performed at the University Hospital Bonn between 2006 and 2017 were analysed retrospectively.” |
|  |  | (*b*) Provide in the abstract an informative and balanced summary of what was done and what was found | “After adapting POSPOM to the German coding system, we were able to validate the score using patient data of a German university hospital. According to previous demonstration for French patient cohorts, we observed a good correlation of POSPOM with in-hospital mortality.” |
| Introduction | | |  |
| Background/rationale | 2 | Explain the scientific background and rationale for the investigation being reported | “Despite the general availability of the required data, the POSPOM has not been validated for the German healthcare system and therefore cannot be routinely used in Germany.” |
| Objectives | 3 | State specific objectives, including any prespecified hypotheses | “It was our aim to enable the application of the POSPOM by adapting it to the national coding system further referred to as G-POSPOM and to validate its prognostic power on data of a large patient sample from a German university hospital.” |
| Methods | | |  |
| Study design | 4 | Present key elements of study design early in the paper | “Adaptation of the POSPOM to the German healthcare coding system was performed by retrospective calculation, based on patient data extracted from the anonymized data set following §21 Krankenhausentgeltgesetz (KHEntgG, German hospital fees act), which is used for billing purposes [14].” |
| Setting | 5 | Describe the setting, locations, and relevant dates, including periods of recruitment, exposure, follow-up, and data collection | “All surgical procedures or interventions involving anaesthesia performed on adult patients (at least 18 years of age) at the University Hospital Bonn, Germany, between January 1st 2006 and December 31st 2017 were identified. Data required for the calculation of the POSPOM were collected by reviewing the institutional §21 KHEntgG electronic database. Investigated endpoints were patients' discharge or in-hospital death. Death after hospital discharge was not taken into account.” |
| Participants | 6 | (*a*) Give the eligibility criteria, and the sources and methods of selection of participants. Describe methods of follow-up | “All surgical procedures or interventions involving anaesthesia performed on adult patients (at least 18 years of age) at the University Hospital Bonn, Germany, between January 1st 2006 and December 31st 2017 were identified. Data required for the calculation of the POSPOM were collected by reviewing the institutional §21 KHEntgG electronic database. Investigated endpoints were patients' discharge or in-hospital death. Death after hospital discharge was not taken into account.” |
|  |  | (*b*) For matched studies, give matching criteria and number of exposed and unexposed | - |
| Variables | 7 | Clearly define all outcomes, exposures, predictors, potential confounders, and effect modifiers. Give diagnostic criteria, if applicable | “Investigated endpoints were patients' discharge or in-hospital death. Death after hospital discharge was not taken into account. The POSPOM variables are included in S1 Table.” |
| Data sources/ measurement | 8* | For each variable of interest, give sources of data and details of methods of assessment (measurement). Describe comparability of assessment methods if there is more than one group | “All surgical procedures or interventions involving anaesthesia performed on adult patients (at least 18 years of age) at the University Hospital Bonn, Germany, between January 1st 2006 and December 31st 2017 were identified. Data required for the calculation of the POSPOM were collected by reviewing the institutional §21 KHEntgG electronic database.” |
| Bias | 9 | Describe any efforts to address potential sources of bias | “French index surgeries were manually assigned to German OPS equivalents. Inconclusive surgical procedures were reviewed by specialist surgeons of the corresponding departments. Patients that underwent multiple surgeries had their first index surgery assigned. In case of a patient having more than one relevant index procedure encoded at the same time, we assigned the surgery scoring the most POSPOM points.” |
| Study size | 10 | Explain how the study size was arrived at | “All surgical procedures or interventions involving anaesthesia performed on adult patients (at least 18 years of age) at the University Hospital Bonn, Germany, between January 1st 2006 and December 31st 2017 were identified.” |
| Quantitative variables | 11 | Explain how quantitative variables were handled in the analyses. If applicable, describe which groupings were chosen and why |  |
| Statistical methods | 12 | (*a*) Describe all statistical methods, including those used to control for confounding | “Statistical performance and accuracy of the prognostic model was measured testing discrimination and calibration. Discrimination was checked and visualized by a receiver operating characteristic (ROC) analysis and calculation of its area under the curve (AUC), also termed c-statistic [20]. Possible values of the AUC vary from 0.5 (no predictive ability) to 1.0 (perfect predictive ability). The Brier score assesses the overall accuracy (discrimination and calibration), ranging from 0 (implying perfect prediction) to 1 (worst possible prediction). Calibration was visualized via a calibration plot in which a gradient of 1 with the diagonal crossing the origin equals perfect calibration.  All analyses were carried out using the software R (Version 3.5.0 (http://www.r-project.org), last date accessed: January 23, 2020) under creative common license and the affiliated packages ggplot2, dplyr, and pROC [21-23].” |
|  |  | (*b*) Describe any methods used to examine subgroups and interactions |  |
|  |  | (*c*) Explain how missing data were addressed | “Patients were excluded from analyses if any of the POSPOM variables were not attainable for a patient during the investigation period.” |
|  |  | (*d*) If applicable, explain how loss to follow-up was addressed | - |
|  |  | (*e*) Describe any sensitivity analyses | “Statistical performance and accuracy of the prognostic model was measured testing discrimination and calibration. Discrimination was checked and visualized by a receiver operating characteristic (ROC) analysis and calculation of its area under the curve (AUC), also termed c-statistic [20]. Possible values of the AUC vary from 0.5 (no predictive ability) to 1.0 (perfect predictive ability). The Brier score assesses the overall accuracy (discrimination and calibration), ranging from 0 (implying perfect prediction) to 1 (worst possible prediction). Calibration was visualized via a calibration plot in which a gradient of 1 with the diagonal crossing the origin equals perfect calibration.” |
| Results | | |  |
| Participants | 13* | (a) Report numbers of individuals at each stage of study—eg numbers potentially eligible, examined for eligibility, confirmed eligible, included in the study, completing follow-up, and analysed | “A total of 357,861 surgical cases during the time period between January 1st 2006 and December 31st 2017 were identified from the institutional data base at the University Hospital Bonn. Of those cases, 115,281 had no index procedures relevant for the POSPOM and were therefore excluded from further analyses. These were mainly interventions during intensive care therapy, patients undergoing electroconvulsive treatment, or patients having received minor interventions such as biopsies. 41,836 cases were excluded as the patients were younger than 18 years. Finally, 964 cases showed an incomplete dataset with at least one missing relevant information, resulting in a POSPOM not being able to be calculated. Figure 1 shows the patient flow chart diagram.” |
|  |  | (b) Give reasons for non-participation at each stage |  |
|  |  | (c) Consider use of a flow diagram |  |
| Descriptive data | 14* | (a) Give characteristics of study participants (eg demographic, clinical, social) and information on exposures and potential confounders | “A total of 357,861 surgical cases during the time period between January 1st 2006 and December 31st 2017 were identified from the institutional data base at the University Hospital Bonn. Of those cases, 115,281 had no index procedures relevant for the POSPOM and were therefore excluded from further analyses. These were mainly interventions during intensive care therapy, patients undergoing electroconvulsive treatment, or patients having received minor interventions such as biopsies. 41,836 cases were excluded as the patients were younger than 18 years. Finally, 964 cases showed an incomplete dataset with at least one missing relevant information, resulting in a POSPOM not being able to be calculated. Figure 1 shows the patient flow chart diagram.” |
|  |  | (b) Indicate number of participants with missing data for each variable of interest |  |
|  |  | (c) Summarise follow-up time (eg, average and total amount) | - |
| Outcome data | 15* | Report numbers of outcome events or summary measures over time | “We included a total of 199,780 patient cases along with 4,053 in-hospital deaths into our study, resulting in a hospital mortality of 2.04% (95% CI 1.97 to 2.09%).” |
| Main results | 16 | (*a*) Give unadjusted estimates and, if applicable, confounder-adjusted estimates and their precision (eg, 95% confidence interval). Make clear which confounders were adjusted for and why they were included | “We included a total of 199,780 patient cases along with 4,053 in-hospital deaths into our study, resulting in a hospital mortality of 2.04% (95% CI 1.97 to 2.09%). In our cohort 98,376 patients (49.24%) were female and 101,394 patients (50.75%) were male with an in-hospital mortality of 1.73% and 2.33% respectively. The highest score value observed was 49 POSPOM-Points. Mean POSPOM value in our cohort was 18.18 (SD 8.11), the median POSPOM was 18 points. Women had a mean POSPOM of 16.96 (SD 7.90) and men a higher mean of 19.35 (SD 8.14). Mean age was 56.33 (SD 18.59) years, and the median age was 59 years. Mean age of male patients was 58.38 (SD 17.45) years, while the median age was 61 years. Woman had a mean age of 54.25 (SD 19.47) years and a median age of 55 years. In total, 3,631,032 POSPOM points were applied to our patient cohort. Of those, 354,229 points (9.76%) were scored for comorbidities, 1,531,337 points (42.17%) for age, and 1,745,466 points (48.07%) were scored for surgeries.” |
|  |  | (*b*) Report category boundaries when continuous variables were categorized | - |
|  |  | (*c*) If relevant, consider translating estimates of relative risk into absolute risk for a meaningful time period | - |
| Other analyses | 17 | Report other analyses done—eg analyses of subgroups and interactions, and sensitivity analyses | “We included a total of 199,780 patient cases along with 4,053 in-hospital deaths into our study, resulting in a hospital mortality of 2.04% (95% CI 1.97 to 2.09%). In our cohort 98,376 patients (49.24%) were female and 101,394 patients (50.75%) were male with an in-hospital mortality of 1.73% and 2.33% respectively. The highest score value observed was 49 POSPOM-Points. Mean POSPOM value in our cohort was 18.18 (SD 8.11), the median POSPOM was 18 points. Women had a mean POSPOM of 16.96 (SD 7.90) and men a higher mean of 19.35 (SD 8.14). Mean age was 56.33 (SD 18.59) years, and the median age was 59 years. Mean age of male patients was 58.38 (SD 17.45) years, while the median age was 61 years. Woman had a mean age of 54.25 (SD 19.47) years and a median age of 55 years. In total, 3,631,032 POSPOM points were applied to our patient cohort. Of those, 354,229 points (9.76%) were scored for comorbidities, 1,531,337 points (42.17%) for age, and 1,745,466 points (48.07%) were scored for surgeries.” |
| Discussion | | |  |
| Key results | 18 | Summarise key results with reference to study objectives | “Our study demonstrates that the POSPOM, originally derived from French data, can successfully be transferred into other national healthcare systems, in our case by matching to the German OPS coding. Thereby the adapted G-POSPOM may accurately predict postoperative in-hospital mortality.” |
| Limitations | 19 | Discuss limitations of the study, taking into account sources of potential bias or imprecision. Discuss both direction and magnitude of any potential bias | “The present study is a retrospective, single-centered analysis integrating data recorded over a period of 11 years and is therefore limited in its comparability to the French multi-centre derivation cohort assessed within one year.”  “The POSPOM does not distinguish between emergency or elective surgery. However, seen from the clinician’s point of view, there is obviously a considerable difference between elective and emergency patients, regarding mortality rate. Therefore, it might be reasonable to take the treatment mode such as elective, urgent or even emergent into account for the prognostic model and thus for scoring.” |
| Interpretation | 20 | Give a cautious overall interpretation of results considering objectives, limitations, multiplicity of analyses, results from similar studies, and other relevant evidence | “In summary, in our analysis the G-POSPOM proved to be a valuable score to predict in-hospital mortality in patients undergoing elective as well as emergency surgery. Using preoperatively available data from a retrospective data base record, we could demonstrate that the POSPOM is applicable to the German healthcare system as well as to the patient population of a single-centre university hospital. However, further multicentric validation is mandatory as the patient population of a single university hospital most likely does not represent the overall German healthcare system. Recalibration and prospective study designs could help to further improve the POSPOM’s applicability to the German patient population as much as to other national healthcare systems.” |
| Generalisability | 21 | Discuss the generalisability (external validity) of the study results |  |
| Other information | | |  |
| Funding | 22 | Give the source of funding and the role of the funders for the present study and, if applicable, for the original study on which the present article is based | “The authors received no specific funding for this work.” |

*Give information separately for exposed and unexposed groups.

**Note:** An Explanation and Elaboration article discusses each checklist item and gives methodological background and published examples of transparent reporting. The STROBE checklist is best used in conjunction with this article (freely available on the Web sites of PLoS Medicine at http://www.plosmedicine.org/, Annals of Internal Medicine at http://www.annals.org/, and Epidemiology at http://www.epidem.com/). Information on the STROBE Initiative is available at http://www.strobe-statement.org.
