# Supplementary material for: Validation of the Preoperative Score to Predict Postoperative Mortality (POSPOM) in Germany
Source: PLoS One. 2021 Jan 27;16(1):e0245841. doi: 10.1371/journal.pone.0245841 (PMC7840059; doi:10.1371/journal.pone.0245841)
Supplement: S3 Table — (DOCX) [file pone.0245841.s003.docx]

| G-POSPOM | N | Percentage of cohort (%) | In-hospital deceased | Mortality (%) |
| --- | --- | --- | --- | --- |
| 0 | 372 | 0.186 | 0 | 0 |
| 1 | 635 | 0.318 | 1 | 0.157 |
| 2 | 644 | 0.322 | 0 | 0 |
| 3 | 641 | 0.321 | 2 | 0.312 |
| 4 | 865 | 0.433 | 3 | 0.347 |
| 5 | 1270 | 0.636 | 9 | 0.709 |
| 6 | 3156 | 1.580 | 8 | 0.253 |
| 7 | 5328 | 2.667 | 8 | 0.150 |
| 8 | 8130 | 4.069 | 21 | 0.258 |
| 9 | 11841 | 5.927 | 19 | 0.160 |
| 10 | 12054 | 6.034 | 37 | 0.307 |
| 11 | 9577 | 4.794 | 39 | 0.407 |
| 12 | 8644 | 4.327 | 46 | 0.532 |
| 13 | 7674 | 3.841 | 75 | 0.977 |
| 14 | 7252 | 3.630 | 72 | 0.993 |
| 15 | 6861 | 3.434 | 82 | 1.195 |
| 16 | 6749 | 3.378 | 65 | 0.963 |
| 17 | 6611 | 3.309 | 65 | 0.983 |
| 18 | 6716 | 3.362 | 78 | 1.161 |
| 19 | 7064 | 3.536 | 82 | 1.161 |
| 20 | 7645 | 3.827 | 106 | 1.387 |
| 21 | 7806 | 3.907 | 127 | 1.627 |
| 22 | 7533 | 3.771 | 149 | 1.978 |
| 23 | 7178 | 3.593 | 169 | 2.354 |
| 24 | 7066 | 3.537 | 190 | 2.689 |
| 25 | 7190 | 3.599 | 213 | 2.962 |
| 26 | 7333 | 3.671 | 239 | 3.259 |
| 27 | 6604 | 3.306 | 263 | 3.982 |
| 28 | 5571 | 2.789 | 252 | 4.523 |
| 29 | 4927 | 2.466 | 245 | 4.973 |
| 30 | 4097 | 2.051 | 260 | 6.346 |
| 31 | 3536 | 1.770 | 231 | 6.533 |
| 32 | 2954 | 1.479 | 219 | 7.414 |
| 33 | 2362 | 1.182 | 177 | 7.494 |
| 34 | 1975 | 0.989 | 140 | 7.089 |
| 35 | 1448 | 0.725 | 120 | 8.287 |
| 36 | 920 | 0.461 | 91 | 9.891 |
| 37 | 619 | 0.310 | 56 | 9.047 |
| 38 | 388 | 0.194 | 44 | 11.340 |
| 39 | 247 | 0.124 | 23 | 9.312 |
| 40 | 147 | 0.074 | 27 | 18.367 |
| 41 | 73 | 0.037 | 2 | 2.740 |
| 42 | 37 | 0.019 | 7 | 18.919 |
| 43 | 17 | 0.009 | 1 | 5.882 |
| 44 | 12 | 0.006 | 1 | 8.333 |
| 45 | 6 | 0.003 | 1 | 16.667 |
| 46 | 2 | 0.001 | 1 | 50 |
| 47 | 0 | 0 | 0 | - |
| 48 | 2 | 0.001 | 0 | 0 |
| 49 | 1 | 0.001 | 0 | 0 |
