# Supplementary material for: Validation of the Preoperative Score to Predict Postoperative Mortality (POSPOM) in Germany
Source: PLoS One. 2021 Jan 27;16(1):e0245841. doi: 10.1371/journal.pone.0245841 (PMC7840059; doi:10.1371/journal.pone.0245841)
Supplement: S4 Table — (DOCX) [file pone.0245841.s004.docx]

| Year | Mortality | POSPOM mean (SD) |
| --- | --- | --- |
| 2006 | 1.71 | 18.31 (7.92) |
| 2007 | 1.80 | 18.19 (7.93) |
| 2008 | 1.84 | 18.07 (7.90) |
| 2009 | 2.00 | 17.42 (7.79) |
| 2010 | 1.95 | 17.71 (7.96) |
| 2011 | 1.77 | 17.81 (8.04) |
| 2012 | 1.94 | 18.15 (8.08) |
| 2013 | 2.05 | 18.16 (8.20) |
| 2014 | 2.07 | 18.16 (8.26) |
| 2015 | 2.14 | 18.89 (8.19) |
| 2016 | 2.64 | 18.93 (8.36) |
| 2017 | 2.52 | 18.90 (8.53) |
