# Supplementary material for: Validation of the Preoperative Score to Predict Postoperative Mortality (POSPOM) in Germany
Source: PLoS One. 2021 Jan 27;16(1):e0245841. doi: 10.1371/journal.pone.0245841 (PMC7840059; doi:10.1371/journal.pone.0245841)
Supplement: S5 Table — (DOCX) [file pone.0245841.s005.docx]

| Demographics |  |  |  |  |
| --- | --- | --- | --- | --- |
| Age (yr; mean, SD, Median) | 56.33 | 18.59 | 59 |  |
| Male sex (%, n) | 50.75 | 101,394 |  |  |
|  |  |  |  |  |
| Medical history | N= | In-hospital death= | Mortality (%) | Proportion (%) |
| Ischemic Heart Disease | 23,088 | 1,306 | 5.66 | 11.56 |
| Cardiac arrythmia or heart block | 10,321 | 789 | 7.64 | 5.17 |
| Peripheral vascular disease or abdominal aortic aneurysm | 9,772 | 686 | 7.02 | 4.89 |
| Cerebrovascular Disease | 2,468 | 117 | 4.74 | 1.24 |
| Chronic obstructive pulmonary disease | 7,586 | 474 | 6.25 | 3.80 |
| Diabetes | 19,624 | 848 | 4.32 | 9.82 |
| Preoperative chronic hemodialysis | 1,352 | 119 | 8.80 | 0.68 |
| Dementia | 1,984 | 141 | 7.11 | 0.99 |
| Transplanted organs | 1,974 | 88 | 4.46 | 0.99 |
| Chronic renal failure | 10,659 | 712 | 6.68 | 5.34 |
| Chronic respiratory failure | 656 | 67 | 10.21 | 0.33 |
| Chronic heart failure or cardiomyopathy | 8,702 | 411 | 4.72 | 4.36 |
| Hemiplegia | 7,846 | 501 | 6.39 | 3.93 |
| Chronic alcohol abuse | 4,544 | 286 | 6.29 | 2.27 |
| Cancer | 41,112 | 1191 | 2.90 | 20.58 |
