# Supplementary material for: Validation of the Preoperative Score to Predict Postoperative Mortality (POSPOM) in Germany
Source: PLoS One. 2021 Jan 27;16(1):e0245841. doi: 10.1371/journal.pone.0245841 (PMC7840059; doi:10.1371/journal.pone.0245841)
Supplement: S6 Table — (DOCX) [file pone.0245841.s006.docx]

| Surgical procedures |  |  |
| --- | --- | --- |
|  | N= | Median POSPOM= |
| Cardiac surgery | 10,887 | 32 |
| Interventional Neuroradiology | 3,047 | 27 |
| Interventional Cardiorhytmology | 3,562 | 22 |
| Urologic surgery | 9,103 | 21 |
| Vascular surgery | 6,769 | 26 |
| Plastic surgery | 13,760 | 20 |
| Ophtalmology | 22,304 | 10 |
| Gynecologic surgery | 20,694 | 9 |
| Neurosurgery | 21,705 | 25 |
| Ear, nose and throat (ENT) surgery | 23,124 | 16 |
| Orthopedic surgery | 24,855 | 16 |
| Transplant surgery | 395 | 32 |
| Digestive surgery | 13,104 | 24 |
| Liver, biliary tract and pancreas surgery | 3,891 | 25 |
| Thoracic surgery | 2,028 | 27 |
| Endoscopy | 15,204 | 11 |
| Others | 5,348 | 24 |
| Total | 199,780 | 18 |
